# LFPy: a tool for biophysical simulation of extracellular potentials generated by detailed model neurons

**DOI:** 10.3389/fninf.2013.00041

**Published:** 2014-01-16

**Authors:** Henrik Lindén, Espen Hagen, Szymon Łęski, Eivind S. Norheim, Klas H. Pettersen, Gaute T. Einevoll

**Affiliations:** ^1^Department of Mathematical Sciences and Technology, Norwegian University of Life SciencesÅs, Norway; ^2^Department of Computational Biology, School of Computer Science and Communication, Royal Institute of Technology (KTH)Stockholm, Sweden; ^3^Department of Neurophysiology, Nencki Institute of Experimental BiologyWarsaw, Poland; ^4^CIGENE, Norwegian University of Life SciencesÅs, Norway

**Keywords:** local field potential, extracellular potential, biophysics, forward modeling, compartmental modeling, detailed morphology, spike waveform, Python

## Abstract

Electrical extracellular recordings, i.e., recordings of the electrical potentials in the extracellular medium between cells, have been a main work-horse in electrophysiology for almost a century. The high-frequency part of the signal (≳500 Hz), i.e., the *multi-unit activity (MUA)*, contains information about the firing of action potentials in surrounding neurons, while the low-frequency part, the *local field potential (LFP)*, contains information about how these neurons integrate synaptic inputs. As the recorded extracellular signals arise from multiple neural processes, their interpretation is typically ambiguous and difficult. Fortunately, a precise biophysical modeling scheme linking activity at the cellular level and the recorded signal has been established: the extracellular potential can be calculated as a weighted sum of all transmembrane currents in all cells located in the vicinity of the electrode. This computational scheme can considerably aid the modeling and analysis of MUA and LFP signals. Here, we describe **LFPy**, an open source Python package for numerical simulations of extracellular potentials. **LFPy** consists of a set of easy-to-use classes for defining cells, synapses and recording electrodes as Python objects, implementing this biophysical modeling scheme. It runs on top of the widely used NEURON simulation environment, which allows for flexible usage of both new and existing cell models. Further, calculation of extracellular potentials using the line-source-method is efficiently implemented. We describe the theoretical framework underlying the extracellular potential calculations and illustrate by examples how **LFPy** can be used both for simulating LFPs, i.e., synaptic contributions from single cells as well a populations of cells, and MUAs, i.e., extracellular signatures of action potentials.

## 1. Introduction

A host of experimental techniques are now available for studies of neural activity in cortex. In addition to intracellular and extracellular recordings with various types of single- or multi-contact electrodes, several imaging techniques (e.g., two-photon calcium, intrinsic optical, voltage-sensitive dye) have been developed and refined in the last decade. To take full advantage of these new powerful techniques, proper links between the underlying neural activity and what is recorded in the experiments, must be established. Such quantitatively accurate links generally require detailed understanding of the underlying physics of neural activity measurements, as well as efficient mathematical modeling schemes (Brette and Destexhe, 2012; Panzeri and Quian Quiroga, 2013). Computational neuroscience has until now largely focused on how neurons and neural networks may process information, while less attention has been given to the relationship between the neural activity and measurable quantities. As the ultimate test of candidate theories in all natural sciences is comparison with experiments, we believe more focus on the latter is needed to bring the field forward. Not only must precise mathematical links between activity in neural networks and the various measurements be forged, efficient and easy-to-use neuroinformatics tools must be developed to facilitate such comparisons.

The present paper describes a step toward this goal, that is, a new Python-based tool, LFPy (compneuro.umb.no/LFPy, software.incf.org/software/lfpy), for modeling of extracellular potentials stemming from neural activity in brain tissue. While extracellular electrical recordings have been the main workhorse in electrophysiology for almost a century, the interpretation of such recordings is not trivial. The recorded extracellular potentials in general arise from a complicated sum of contributions from all transmembrane currents of the cells, predominantly neurons, in the vicinity of the electrode contact. The high-frequency part of the signal (≳500 Hz), the *multi-unit activity* (MUA), contains information about the firing of action potentials of neurons within a few tens of micrometers or so from the electrode contact (Buzsáki, 2004; Pettersen and Einevoll, 2008). The low-frequency part, the *local field potential* (LFP), contains information about the integration of synaptic inputs in populations of neurons within radii of hundreds of micrometers or more (Lindén et al., 2011; Łęski et al., 2013). Both the MUA and LFP are difficult signals to interpret (Pettersen et al., 2008; Buzsáki et al., 2012; Einevoll et al., 2012, 2013a,b; Reimann et al., 2013), and in order to take full advantage of the new generation of silicon-based multielectrodes recording from tens or hundreds of positions simultaneously, we need to develop and validate methods that can be used to infer information about the underlying neural network from these extracellular signals (Einevoll et al., 2013a).

A key advantage compared to other important measures of neural activity, such as fMRI, is that the ‘measurement physics’, i.e., the link between neural activity and what is measured, is well-understood. In fact, the last decade has seen the refinement of a well-founded biophysical forward-modeling scheme based on volume conduction theory (Rall and Shepherd, 1968; Holt and Koch, 1999) to incorporate detailed reconstructed neuronal morphologies in precise calculations of extracellular potentials — both spikes (Holt and Koch, 1999; Gold et al., 2006, 2007; Pettersen and Einevoll, 2008; Pettersen et al., 2008; Schomburg et al., 2012; Reimann et al., 2013), and LFPs (Einevoll et al., 2007; Pettersen et al., 2008; Lindén et al., 2010, 2011; Gratiy et al., 2011; Schomburg et al., 2012; Łęski et al., 2013; Reimann et al., 2013). The word ‘forward’ denotes that the extracellular potentials are modeled from known neural sources (as opposed to the ill-posed “inverse” problem of estimating the underlying sources from recorded potentials). According to the linear volume conduction theory, the extracellular potentials at the electrode contact can be calculated by adding contributions from the transmembrane currents (Nunez and Srinivasan, 2006). In a now frequently used two-step computational scheme, morphologically reconstructed neurons are first simulated with compartmental modeling using a simulation program such as NEURON (Carnevale and Hines, 2006; Carnevale, 2007) to provide transmembrane currents, and next the extracellular potentials are calculated based on these (Holt and Koch, 1999; Pettersen et al., 2012); (Einevoll et al., 2013a,b).

**Figure 1:**
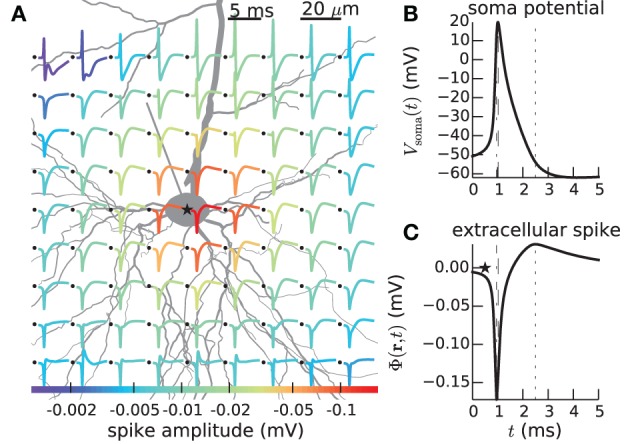
**Calculated extracellular spike waveforms using LFPy. (A)** Position-dependent extracellular spike waveforms during an action potential in a rat L5b pyramidal-cell model (Hay et al., 2011) produced by executing example2.py, see section 4.2. Black dots correspond to the positions of the (virtual) electrode contact points. Spike traces at each position are normalized and color coded according to the magnitude of the negative peak. **(B)** Corresponding somatic membrane potential during the action potential. Vertical dashed lines illustrate temporal alignment with the maximum magnitude *V*_soma_(*t*) and of an extracellular waveform **(C)** for the position denoted with an asterisk in **(A)**. Corresponding alignment with the maximum positive extracellular peak is illustrated by vertical dotted lines.

In LFPy these two steps are performed in an integrated Python environment employing the NEURON simulator (Carnevale and Hines, 2006; Carnevale, 2007; Hines et al., 2009) under the hood, allowing full advantage of the plethora of packages available for the Python programming language. For example, existing multicompartmental neuron models, available from databases like ModelDB^1^ (Hines et al., 2004), can readily be adapted for use with the LFPy-package. An example of such analysis made simple with LFPy is shown in Figure 1, showing a spike, i.e., the extracellular signature of an action potential, from simulations using a recently published rat L5b model neuron by Hay et al. (2011) with LFPy. While the first released version of LFPy described here focuses on calculations of extracellular potentials around individual neurons, the tool is directly applicable also to populations of many such individual neurons, that is, model populations for which the synaptic input onto each neuron is described explicitly and do not necessarily follow from concurrent network simulations (Lindén et al., 2011; Łęski et al., 2013).

The paper is organized as follows: In section 2 we briefly review the biophysics underlying the forward-modeling scheme used in LFPy, in section 3 we give an overview over the different Python class-objects in LFPy, in section 4 we show several examples of the use of LFPy, more technical aspects of the package are described in section 5, while some concluding remarks are given in the final section 6.

## 2. Biophysics behind LFP_y_

Extracellular potentials recorded inside the brain are generated by transmembrane currents from cells in the vicinity of electrode. To propagate from the membrane to the recording electrode, the signal has to pass through brain tissue consisting of a tightly packed matrix of neurons and glial cells embedded in a low-resistance extracellular medium filling less than one fifth of the total volume (Nunez and Srinivasan, 2006). The low resistance of the extracellular medium ensures that neighboring cells are typically electrically decoupled. Further, the difference between the extracellular potentials recorded at different positions will be small, typically much smaller than a millivolt, i.e., about two orders of magnitude smaller than the potential difference across the highly resistant cell membranes.

The biophysical origin of the recorded signals is fortunately quite well-understood, and a well-founded computational scheme has been developed for the forward modeling of the signals. This scheme naturally divides into two consecutive steps: calculation of transmembrane currents stemming from activity in neurons (and glia cells, if relevant) followed by calculation of the extracellular potentials generated by these transmembrane currents. The calculations of extracellular potentials as implemented in LFPy are thus also organized in two steps:

1. Calculation of transmembrane currents of each neuron, using multicompartmental model neurons derived from detailed morphological reconstructions of neurons within NEURON simulation environment (Carnevale and Hines, 2006; Carnevale, 2007).
2. Calculation of the extracellular potential from the transmembrane currents using a biophysical forward-modeling formalism derived within so called *volume-conductor theory* (Hämäläinen et al., 1993; Nunez and Srinivasan, 2006).

### 2.1. Multicompartmental modeling of transmembrane currents

**Figure 2:**
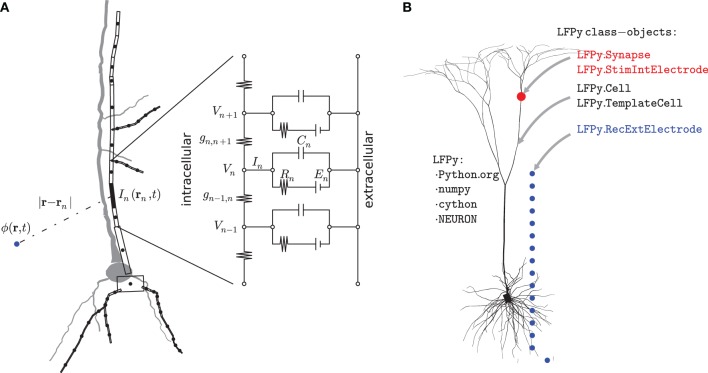
**Illustration of biophysical modeling scheme and corresponding organization of LFPy-package. (A)** Principle of multicompartmental modeling where a piece of an apical dendritic branch, in this example assumed purely passive with only capacitive and leak membrane currents, is divided into a set of compartments indexed by *n*. The circuit diagram shows the equivalent electric circuit of the compartment where the dynamics is governed by an equation set consisting of equations of the type shown in Equation (1). The net transmembrane current *I_n_(t)*, in this case the sum over the capacitive and leak membrane currents in compartment *n*, is then used in the forward-modeling schemes in Equations (3–5) to calculate extracellular potentials. **(B)** LFPy is a Python package, dependent on numpy, Cython and NEURON, implementing this combined modeling scheme. Its main features are object-oriented representations of biophysically detailed neurons (LFPy.Cell, LFPy.TemplateCell), point-process mechanisms such as synapses or patch electrodes attached to different locations of the cell-objects (LFPy.Synapse, LFPy.StimIntElectrode), and an extracellular recording device allowing calculation of extracellular potentials at arbitrary locations (LFPy.RecExtElectrode).

In the first step, multicompartment neuron models are used to calculate transmembrane currents. Figure 2A illustrates the principle behind the construction of such multicompartmental models where the neuron is divided into compartments, each so small that the electrical potential can be assumed to be the same throughout the compartment (Segev et al., 1989; De Schutter, 2009). Every compartment is described as an equivalent electrical circuit where the key dynamical variable is the membrane potential *V*, and the equation describing the dynamics of this variable follows from Kirchhoff's current law stating that the currents going into a circuit node have to sum to zero. For the case where the extracellular potential is assumed constant, the mathematical equation describing the temporal development of the membrane potential *V_n_* of compartment *n* in Figure 2A is given by

(1) $$g_{n,\;n\;+\;1}(V_{n\;+\;1}-V_{n})-g_{n\;-\;1,\;n}(V_{n}-V_{n-1})=C_{n}\frac{dV_{n}}{dt}+\sum_{j}I_{n}^{j}\;\;\;$$

The two terms on the left hand side of the equation represent intracellular ohmic currents between compartment *n* and the neighboring compartments *n* + 1 and *n* − 1. The first term on the right hand side represents currents due to capacitive properties of the cell membrane, while the second term represents currents due to various other membrane processes such as passive and active intrinsic ion channels and synaptic inputs. In the full multicompartmental model of a neuron there will be an equation of the type shown in Equation (1) for each compartment, and the equation set is solved numerically using dedicated simulation tools such as NEURON (Carnevale and Hines, 2006; Carnevale, 2007). The transmembrane current from each neuronal compartment *n* is then at each instant in time given by the right hand side of Equation (1), denoted by *I_n_* in Figure 2A. There are different numerical strategies in terms of the spatial discretization of neuronal models, and in NEURON, the spatial discretization is equivalent to assuming that the transmembrane current density is uniformly distributed in each compartment so that second order accurate intracellular potentials between nodes can be found by linear interpolation (Hines and Carnevale, 2001; Carnevale and Hines, 2006).

Note that Kirchhoff's current law implies that the net transmembrane currents (including the capacitive current) coming out of a neuron at all times must equal zero. Thus with the neuron depicted in Figure 2A divided into *N* compartments, one must at all times have $\sum_{n=1}^{N}I_{n}(t)=0$. Therefore a single-compartment neuron model cannot generate any extracellular potential since the net transmembrane current necessarily will be zero. The simplest model producing an extracellular potential is a two-compartment model where a transmembrane current entering the neuron at one compartment leaves at the other compartment. The simplest possible multipole configuration is thus the current *dipole* (Pettersen et al., 2012).

### 2.2. From transmembrane currents to extracellular potentials

Given the numerical value and spatial position of all transmembrane currents, the extracellular potentials can be computed on the basis of *volume conductor theory*. Here the system can be envisioned as a three-dimensional smooth extracellular continuum with the transmembrane currents represented as *volume current sources* (Nunez and Srinivasan, 2006). In this framework the fundamental relationship between an extracellular potential ϕ(*t*) recorded at position **r** due to a transmembrane current *I*_0_(*t*) at position **r**_0_ is given by (Hämäläinen et al., 1993; Nunez and Srinivasan, 2006):

(2) $$\phi (r,\;t)=\frac{1}{4\pi \sigma }\frac{I_{0}(t)}{\left|r-r_{0}\right|}\;.$$

Here the extracellular potential ϕ is set to be zero infinitely far away from the transmembrane current, and σ is the *extracellular conductivity*, assumed to be *real*, *scalar* (the same in all directions) and *homogeneous* (the same everywhere in an infinite volume conductor). Equation (2) relies on two key assumptions:

1. The *quasistatic approximation* of Maxwell's equations amounting to omitting terms with time derivatives of the electric (**E**) or magnetic fields (**B**) so that these field effectively decouple. For the frequencies inherent in neural activity, i.e., less than a few thousand hertz, this approximation seems to be well-justified [see, e.g., argument on p. 426 in Hämäläinen et al. (1993)].
2. The assumption of a *linear*, *isotropic*, *homogeneous* and *ohmic* extracellular medium, i.e., a linear relationship between the current density **j** and the electrical field **E**, **j** = σ**E**, where σ is a real scalar. The absence of an imaginary part of the conductivity σ implies that the capacitive effects of the extracellular tissue are assumed to be negligible compared to resistive effects. This appears to be well-fulfilled for the relevant frequencies in extracellular recordings (Nunez and Srinivasan, 2006; Logothetis et al., 2007). The fact that σ is a scalar reflects the assumption of isotropic and homogeneous medium.

Note that while the present version of LFPy is based on these assumptions, the forward model in Equation (2) can be generalized to account for, for example, different conductivities in different directions (Nicholson and Freeman, 1975; Logothetis et al., 2007), discontinuities in conductivity at interfaces between gray and white matter or between the gray matter and the cortical surface (Pettersen et al., 2006), or, if warranted, frequency-dependent and/or complex extracellular conductivities σ (Bédard et al., 2004; Bédard and Destexhe, 2012). For more discussion of the validity and possible generalizations of the present forward-modeling scheme, see Pettersen et al. (2012).

Equation (2) applies to the situation with a single current *I*_0_, but since contributions from several transmembrane current sources add linearly, the equation straightforwardly generalizes to a situation with many transmembrane current sources. With *N point current sources* the formula in Equation (2) generalizes to:

(3) $$\phi (r,\;t)=\frac{1}{4\pi \sigma }\sum_{n\;=\;1}^{N}\frac{I_{n}(t)}{\left|r-r_{n}\right|}\;.$$

With a neuron divided into *N* compartments, the natural use of the formula in Equation (3) is to set **r**_*n*_ at the “mean” position of compartment *n*, e.g., at the center of a spherical soma compartment or the mid-point of a cylindrical dendritic compartment. This scheme corresponds to the so called *point-source* approximation (Holt and Koch, 1999; Pettersen et al., 2008) since all transmembrane currents into the extracellular medium go through a single point. Another scheme, the *line-source* approximation, assumes the transmembrane currents from each cylindrical compartment to be evenly distributed along a line corresponding to the cylinder axis (Holt and Koch, 1999; Pettersen et al., 2008). The analogous formula for the line-source approximation is obtained by integrating Equation (3) along the center-line axis along each compartment (Holt and Koch, 1999; Pettersen et al., 2008):

(4) $$\begin{array}{l} \phi (r,\;t)=\frac{1}{4\pi \sigma }\sum_{n\;=\;1}^{N}I_{n}(t)\int \frac{dr_{n}}{\left|r-r_{n}\right|}\\ =\frac{1}{4\pi \sigma }\sum_{n\;=\;1}^{N}I_{n}(t)\frac{1}{\Delta s_{n}}\log\left|\frac{\sqrt{h_{n}^{2}+\rho_{n}^{2}}-h_{n}}{\sqrt{l_{n}^{2}+\rho_{n}^{2}}-l_{n}}\right|\;. \end{array}$$

Here Δ*s_n_* denotes the length of the compartment, ρ_*n*_ the distance perpendicular to the line compartment, *h*_*n*_ the longitudinal distance from the end of the compartment, and *l*_*n*_ = Δ*s*_*n*_ + *h*_*n*_ the longitudinal distance from the start of the compartment. In this and the above method, singularities are avoided by strictly preventing the denominators (i.e., |**r** − **r**_*n*_|) to be *less* than the radius of the relevant cylindrical compartment. Both the point-source and the line-source approximation schemes are implemented in LFPy, together with a *mixed* method applicable for models with a single-compartment soma as the root section (defined to be compartment 1), effectively treating the soma as a sphere source:

(5) $$\begin{array}{l} \phi (r,\;t)=\frac{1}{4\;\pi \;\sigma }(\frac{I_{1(t)}}{\left|r-r_{1}\right|}\\ +\sum_{n\;=\;2}^{N}I_{n}(t)\frac{1}{\Delta s_{n}}\log\left|\frac{\sqrt{h_{n}^{2}+\rho_{n}^{2}}-h_{n}}{\sqrt{l_{n}^{2}+\rho_{n}^{2}}-l_{n}}\right|)\;. \end{array}$$

The three methods described by Eqs. (3-5) are expected to give similar results in electrode positions far from the cell, while results may differ more for electrode positions close to the neuron's membrane (Holt and Koch, 1999).

Note that the modeling scheme as presented here is not fully self-consistent as the calculation of the transmembrane currents (Equation (1)) is done assuming constant potentials outside the neuron, which corresponds to assuming negligible resistivity in the extracellular medium. In contrast, a finite resistivity (~ 1/σ) is assumed in the forward models. This approximation ensures efficient forward modeling of extracellular potentials allowing, for example, for calculation of LFPs from populations of tens of thousands of neurons (Lindén et al., 2011; Łęski et al., 2013; Reimann et al., 2013).

The forward-modeling formulas in Eqs. (3-5) all predict potentials at points, while real recording electrodes of course have a physical extension. Finite-sized electrode appears to measure the average potential across the uninsulated electrode surface (Nelson and Pouget, 2010), and here we thus approximate the potential recorded by an ideal electrode contact as the average potential across its surface *S* as:

(6) $$\begin{array}{l} \phi (r,\;t;S)=\frac{1}{A_{S}}\iint_{S}\phi (r^{\prime },\;t)d^{2}r^{\prime }\\ \;\approx \frac{1}{m}\sum_{i\;=\;1}^{m}\phi_{i}(r_{i^{\prime }},\;t)\;, \end{array}$$

for *m* random locations **r**′_*i*_ on the surface *S*, with surface area *A_S_*, of the electrode contact. The surface *S* is by LFPy assumed to be flat and circular.

## 3. Overview of LFP_y_

In this section we give a brief overview over the different classes available in LFPy as illustrated in Figure 2B.

### 3.1. Representing cells in LFP_y_

The main class in LFPy is LFPy.Cell. It is used to create objects that represent individual model cells, and it serves as an interface with the NEURON simulation environment (Carnevale and Hines, 2006; Carnevale, 2007; Hines et al., 2009). It is also necessary for interactions with other LFPy class-objects. Each LFPy.Cell-object stores information about the neuron model, most importantly references to all sections (branches) of the neuron and their geometry, and some parameters of the simulation.

When creating a LFPy.Cell object, one can set various properties of the neuron model and specify the simulation. During initialization it will:

1. Load the neuron geometry file,
2. Load additional model-specific files (e.g., defining active channels),
3. Assign biophysical properties and insert channel densities,
4. Split sections into appropriate numbers of compartments,
5. Assign all compartments an index at the global cell level (see Sec. 5.7),
6. Specify the position and orientation of the cell and its compartments in space, and
7. Specify simulation duration and temporal resolution.

The standard way of creating a cell object is thus to call LFPy.Cell(), as in the example in section 4.1 below. However, some NEURON models make use of *templates* (Carnevale and Hines, 2006), Ch. 13), and for such models one has to use the inherited class LFPy.TemplateCell, as described in section 4.2. LFPy.Cell will assign references to different sections in the top-level of the NEURON environment, while LFPy.TemplateCell will assign them to a specific template. Although several LFPy.Cell objects may exist simultaneously in Python, only LFPy.TemplateCell allows for multiple cell representations internally in NEURON. Nevertheless, simulations of networks of cells are currently not supported in LFPy for reasons discussed in section 5.6.

After the cell object is created the user can interact with various methods and attributes implemented in the LFPy.Cell (or LFPy.TemplateCell) object. It is, for example, possible to position the cell, inspect properties of sections and compartments of the cell, and to specify which of the variables (membrane voltage, ionic currents) should be recorded during the simulation. Finally, the simulation is started by calling the simulate() method which initializes and executes the model in NEURON.

### 3.2. Cell stimulation

LFPy provides two classes, LFPy.Synapse and LFPy.StimIntElectrode (cf. red dot in Figure 2B), which can be used to specify inputs to the cell. They manage synaptic currents triggered by input spike trains and intracellular patch-clamp electrodes, respectively, using NEURON point processes, such as ExpSyn and IClamp (but user-defined synapses or stimulation electrodes are possible through the use of .mod-files specified using the NEURON model description language NMODL (Carnevale and Hines, 2006, Ch. 9)). One important difference between synapses and electrodes is that an electrode current is *not* a transmembrane current in that the current does not come from the extracellular space. This implies that the total current across the cell's membrane no longer will sum to zero, resulting in monopole contributions in the extracellular potential (as the injected current is not included when calculating the extracellular potential). Direct specification of inputs via NEURON is possible as in Hines et al. (2009), but the classes provided in LFPy are usually more convenient: they allow for easy placement of stimulation mechanisms at specified dendritic positions and for easy recording of stimulation currents and membrane voltages in the compartments they are attached to.

### 3.3. Extracellular recording electrodes

Extracellular recording electrode contacts are in LFPy represented by the LFPy.RecExtElectrode class (blue dots, Figure 2B). Creating an electrode object allows specification of the extracellular conductivity and arbitrary configurations of electrode contact points in 3D-space, and choosing either point contacts [i.e., employing Equations (3), (4) or (5)] or finite-size electrode contacts [i.e., Equation (6)].

The RecExtElectrode class provides three different methods for calculating the extracellular potential. These are (1) the point-source approximation [Equation (3), keyword argument method=“pointsource”], (2) the line-source approximation [Equation (4), keyword argument method=“linesource”], and (3) the mixed method where the soma section is treated as a point source and dendritic compartments are treated as line sources [Equation (5), keyword argument method=som_as_point]. The last method should only be used if the soma is the root of the morphology and represented by a single compartment. If this is not the case, LFPy erroneously assigns a point current-source to another compartment.

The extracellular potential can be calculated using RecExtElectrode either *after* the simulation using stored recorded membrane currents, or *at run time*, i.e., each time the NEURON simulation advances one time step (see section 5.9). The latter approach avoids the need for storing recorded membrane currents. We illustrate both methods in Examples below.

As the methods for calculating the extracellular potential may be useful outside LFPy, the low-level implementations of Eqs. (3–5) are not contained within RecExtElectrode, but may be used directly by means of any of the functions LFPy.lfpcalc.calc_lfp^*^. They take keyword arguments cell, x, y, z, sigma, respectively a Cell-like instance, extracellular coordinates and extracellular conductivity, and return the extracellular potentials calculated from recorded, transmembrane currents. In section 5.9 we discuss how to use LFPy with other methods for calculating the extracellular potentials, i.e., other forward models, than the ones currently provided and described in section 2.2.

## 4. Examples

We next consider a few simple examples on how LFPy can be used for calculations of extracellular potentials. (For installation instructions and other technical aspects please see section 5, and the online documentation^2^).

### 4.1. Single synaptic input into passive pyramidal cell

We start with a minimal example (example1.py), in which we set up a passive layer-5 pyramidal cell receiving input through a single synapse, run the simulation, and calculate the extracellular potential, similar to the setup used in Lindén et al. (2010).

First we import LFPy and numpy^3^ (Jones et al., 2001):


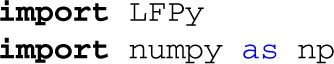


Then we define a dictionary with keyword arguments to be used with LFPy.Cell:


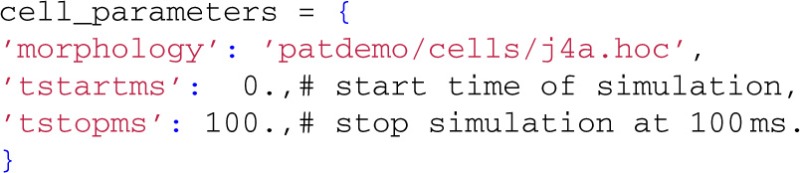


The only mandatory entry is morphology, here pointing to a reconstructed neuron morphology^4^ (Mainen and Sejnowski, 1996) defined with NEURON's HOC-scripting language in a hoc-file. We also specify the start and end times of the simulation (in milliseconds). Several other options are available (such as specifying passive and active parameters of the cell), but for now we leave them at default values.

We are now ready to create our cell instance using the LFPy.Cell-class:


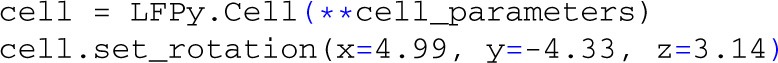


Here we use the cell.set_rotation method to align the apical dendritic branch with the *z*-axis (cf. section 5.3), providing rotation angles in *radians*.

We next attach a synapse to our cell. Again, we define the synapse parameters in a dictionary, and use a method of the cell object to find an appropriate synapse location:


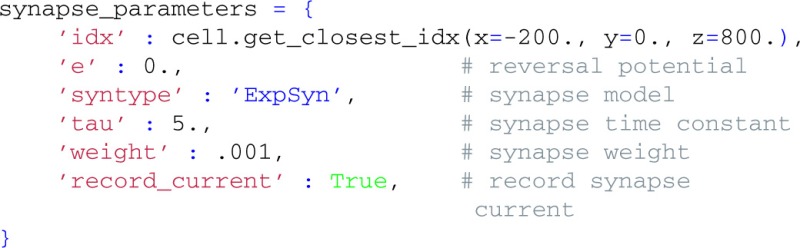


We then create a Synapse object that is connected to our cell by passing cell as an argument, and activate it once at *t* = 20 ms by providing the set_spike_times-method with a numpy-array:


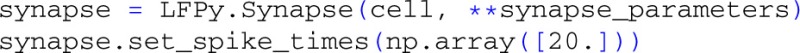


We are now ready to initialize and simulate the postsynaptic response of the cell:


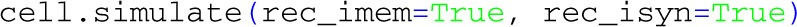


Note the keyword arguments: rec_imem=True sets up the recording of transmembrane current (these are needed later to calculate the extracellular potential), while rec_isyn=True specifies that the synapse current(s) is recorded.

The final step is to set up the extracellular electrode object. Again, we start by defining the parameters,


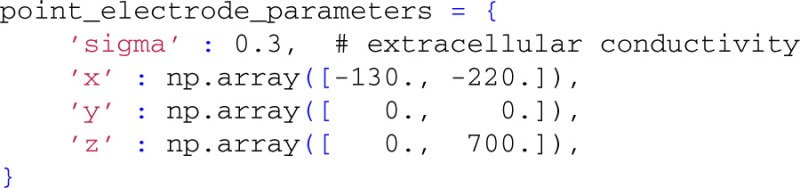


which sets the positions of two extracellular electrode contacts at (*x, y, z*) = (−130, 0, 0) and (−220,0,700) μm, respectively. The number of electrode contact points is defined by the length of the passed arrays containing the contact positions; this allows the user to flexibly define an arbitrary number of recording positions.

By employing class LFPy.RecExtElectrode, we create a Python object representing the extracellular recording devices:


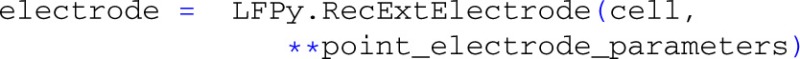


Finally, we calculate the extracellular potential at the specified electrode locations:


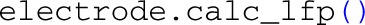


**Figure 3:**
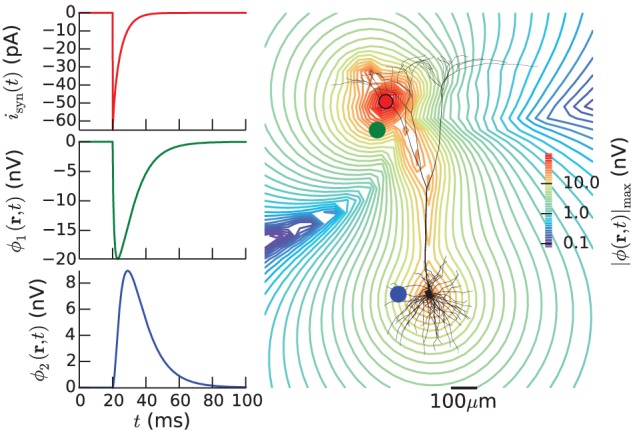
**Extracellular potential generated by a single synaptic input produced by executing example1.py**. Extracellular potentials (**middle** and **bottom left** panels) generated at positions marked by green and blue dot, respectively, by a synaptic input current (**upper left** panel) injected in the apical dendrite at the position marked by red dot. The pyramidal cell corresponds to a layer-5 pyramidal cell from cat visual cortex with passive membranes but without adjustment of the membrane area to compensate for spines (Mainen and Sejnowski, 1996). The contour plot shows equipotential lines for the maximum magnitude of the extracellular potential in the *xz*-plane.

The resulting two extracellular potentials are stored in the numpy-array electrode.LFP, and the results for this example are shown in the left panels of Figure 3. This figure also shows equipotential lines in the *xz*−plane for the maximum potential magnitude, obtained by setting up a second LFPy.RecExtElectrode object representing a grid of recording positions.


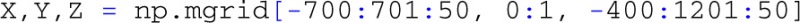


### 4.2. Spiking pyramidal cell with custom hoc code

In the next example the extracellular signature of an action potential generated by a layer-5 pyramidal cell model from Hay et al. (2011) is considered. This example (example2.py), whose outcome is depicted in Figure 1, describes a somewhat more advanced scenario, and serves to illustrate the following features:

1. Use of *network*-ready models with the LFPy.TemplateCell-class,
2. Use of models with active conductances,
3. Use of NeuroLucida V3 morphology file format,
4. Definition of non-zero electrode contact surface area,
5. Calculation of extracellular potentials at run time.

This example requires model files that can be obtained from ModelDB (Hines et al., 2004) (model no. 139653^5^). By unzipping the downloaded model files, all necessary files should be available in the folder L5bPCmodelsEH, including the morphology file and .mod-files describing active membrane mechanisms using NMODL-syntax in L5bPCmodelsEH/mod/. The .mod-files must be compiled with the shell script nrnivmodl (or mknrndll on Windows machines) provided with NEURON, and loaded:


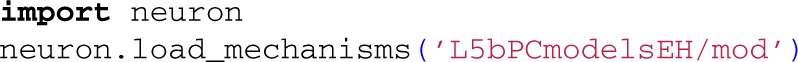


We start by specifying the LFPy.TemplateCell keyword arguments:


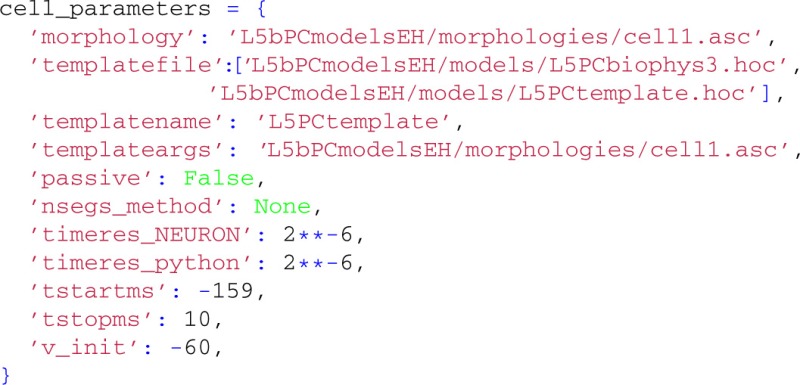


The following keyword arguments are specific to the TemplateCell class:

- templatefile, a (list of) HOC-file(s), specifying the template-file(s) used for the cell object,
- templatename, a string with the name that LFPy.templateCell uses to access the underlying NEURON object, and
- templateargs (optional), that can be used to supply arguments to the template.

Here, we allow the template-files to define the full set of membrane properties as well as the degree of cell compartmentalization [a brief description of template usage are given in section 5.5, but see also (Carnevale and Hines, 2006, Ch. 13)]. The membrane potential is however set to v_init in all sections when the model is initialized, and we override the default values for temporal resolution of the simulation. We are here starting the simulation at *t* = −159 ms to remove a start-up transient and a period of depolarization before the action potential is generated. Recordings of variables and estimations of extracellular potentials will however not occur until the time-step corresponding to *t* = 0 ms is reached.

Since the cell is now defined using a template in NEURON, we employ LFPy.TemplateCell instead of LFPy.Cell to create the cell:


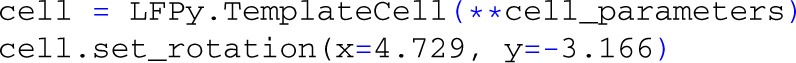


To invoke an action potential in the model, we override the default reversal potential of the passive leak channel originally specified in L5PCbiophys3.hoc:


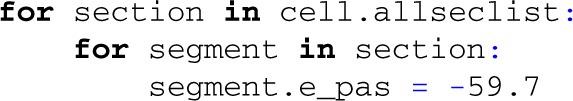


For the extracellular recording device, we simulate the extracellular potentials in a two-dimensional, 9×9 evenly spaced grid with a contact spacing of 20 μm, and specify parameters for contact-surface averaging, including each contact's surface normal vector, common radius and the number of points to include in the estimation of the average potential (cf. Equation 6):


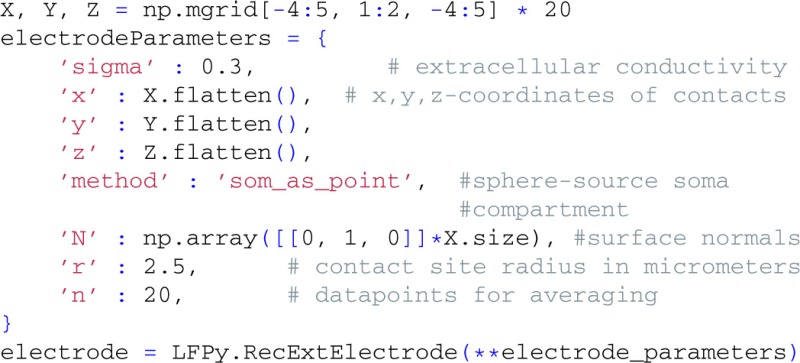


This time we tell LFPy to calculate the extracellular potentials at runtime, by passing the electrode object to cell.simulate:


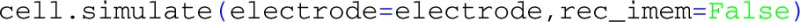


instead of passing cell to the electrode object as we did in the first example above (section 4.1). This assures that the transmembrane currents are discarded after every simulation time step, allowing for more efficient memory usage. Simulation results are shown in Figure 1.

### 4.3. Using MPI for distributed simulations

The present version of LFPy is primarily developed for the study of single cells, but under the present assumption that contributions to the extracellular potential from different cells add linearly (cf. section 2.2), it is straightforward to simulate large populations of cells and sum their contributions. While one option is to simulate cells one after the other, and subsequently sum their contributions, simulations on modern multi-core computers and supercomputers can facilitate greatly from parallelization, e.g., by running computations for different cells on different cores simultaneously. One common way of distributing such simulations is with the Message Passing Interface^6^ (MPI). Below we describe a simple procedure for calculating the extracellular potential from a population of pyramidal cells receiving input from a common pool of presynaptic spike trains. Each neuron is simulated independently, and we rely on MPI to communicate the simulation results of each individual neuron to the root process. This example (example3.py) illustrates how one can easily simulate extracellular potentials of large population of cells similar to the setup used in Lindén et al. (2011) and Łęski et al. (2013) with only a few additional lines of code added to the single cell simulation.

In Python, we will use mpi4py^7^ to interact with the MPI environment:


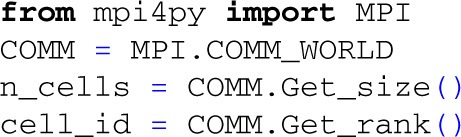


Here, we set the number of cells in the population identical to the number of MPI processes (COMM.Get_size()), and index each cell by the rank of the current process (COMM.Get_rank()). The number of processes is given as an argument to the MPI executable using the -n flag,


mpirun -n 6 python example3.py


which in this case will simulate extracellular potentials from a population of 6 cells, distributed over 6 different Python-processes. Each MPI process will execute the same script, but we can differentiate the instructions to different cells depending on their MPI rank, i.e., with the index returned by COMM.Get_rank(). Note that it is also straightforward to combine serial and parallel execution and to have a constant number of cells in the population, regardless of the number of MPI processes (not shown here).

In the present example we want all cells to share the same pool of presynaptic spike trains, albeit with a different selection of input spike trains for each cell. We first generate the random presynaptic spike trains on each rank, and ensure that the spike-trains are equal for each process by setting the random seed of the numpy random number generator before creating the spike trains:


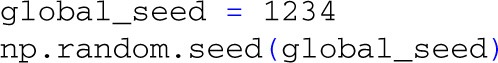


Once the presynaptic spike trains have been created we define the position and rotation for all cells as in previous examples. We then draw a number of presynaptic spike trains for each neuron from the common pool. By setting the number of synapses (each receiving one presynaptic spike train from the pool) for each neuron we can adjust the level of input correlation to the population (Lindén et al., 2011; Łęski et al., 2013). We here choose 100 spike trains for each cell from the pool of 1000 spike trains, giving an input correlation of 0.1 (Lindén et al., 2011). Since these random selections are now done for each cell independently, we assign unique random seeds on each rank:


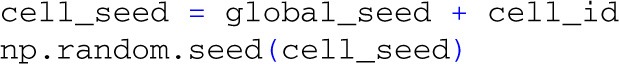


We then create the cell, set up synapses with activation times from the pool of spike trains, and simulate each cell as in the previous example with a single synaptic input (but here with 100 distributed synapses instead of one). Different Python-processes do not yet see each others simulation results, but we may communicate these to the root process (rank zero) using the send, receive and reduce commands provided by MPI:


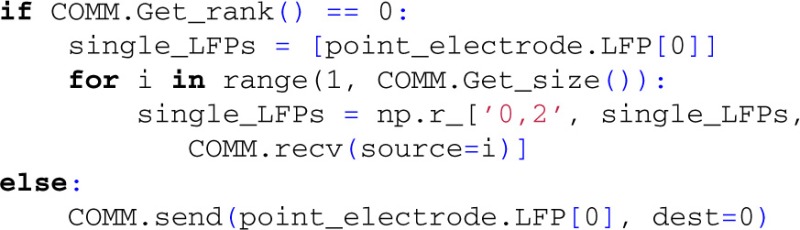


We can also use MPI to sum arrays directly:


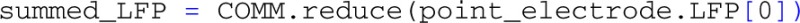


**Figure 4:**
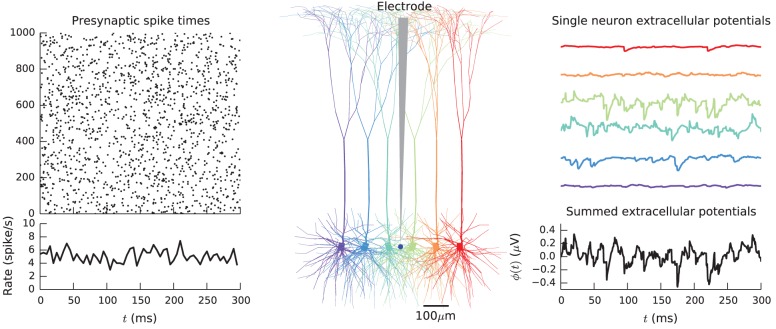
**Simulation of extracellular potentials from a population of neurons using MPI**. A population of pyramidal cells (**Middle** panel) receiving input spikes from a presynaptic pool of spike trains (**Left** panel) is simulated by distributing cells on different MPI processes and by collecting their individual contributions in the root MPI process. Summation of the individual contribution then gives the total population potential (**Right** panel). Results shown come from executing example3.py.

At this point, simulation results have been collected into numpy-arrays on the root process, containing both single-cell and summed contributions to the extracellular potential of the population, as shown in Figure 4.

### 4.4. More examples

Full simulation scripts for reproducing Figure 1 (example2.py) , Figure 3 (example1.py), and Figure 4 (example3.py) can be obtained together with the LFPy source code (ref. section 5.2) in the folder /path/to/LFPy/examples/, along with additional example scripts.

## 5. Technical aspects

### 5.1. Requirements

LFPy is a package for the Python programming language^8^ (Langtangen, 2009), and has primarily been developed and tested on Python 2.7.x, and checked for forward compatibility with Python 3.x. LFPy *should* work on any common 32- or 64-bit platform. The main development and testing platforms have been Debian^9^ derivates and OS X, but LFPy should work equally well on other Unix-like operating systems and Windows. LFPy requires the Python packages NumPy and Cython^10^. Cython allows building C-extensions from comparably slow Python code, which we employed to speed up time-consuming parts of the LFPy codebase, such as the low-level calculations in the line-source method (Holt and Koch, 1999) and the while-loop advancing the simulation time step by time step. The cProfile module^11^ has been used to identify bottlenecks in the code, and the corresponding code was then rewritten using Cython. If Cython is not installed, LFPy will fall back to equivalent but slower Python code. In order to enable all functionality and to run all the example scripts and IPython notebooks successfully, the pylab environment may be required [NumPy, SciPy^12^ (Jones et al., 2001), matplotlib^13^ (Hunter, 2007)], and in addition h5py^14^, mpi4py^15^, IPython^16^ (Pérez and Granger, 2007) version 0.13 or newer with IPython notebook.

LFPy requires the NEURON simulation environment^17^ (Hines and Carnevale, 2001; Carnevale and Hines, 2006) for calculation of the transmembrane currents from activity in multicompartment neuron models. Generic instructions on how to build NEURON as an extension to Python are found in Hines et al., (2009, Appendix), and we maintain some step-by-step instructions for Ubuntu Linux and OS X at the LFPy homepage^18^. Availability of the neuron-module can be checked by issuing:


 python -c “import neuron”
NEURON -- VERSION 7.3 (736+:19ad148877ff+) 19ad148877ff
Duke, Yale, and the BlueBrain Project -- Copyright
 1984-2012
See http://www.neuron.yale.edu/credits.html


While we normally recommend using the standard Python or the interactive IPython interpreters with LFPy, it is in principle possible to use the Python-interpreter supplied with the standard release of NEURON^19^, cf. Section 5.2.3.

### 5.2. Installation and testing

#### 5.2.1. Easy install of LFP_y_

‘Official’ releases of LFPy can be installed in one line from the Python Package index^20^, using either easy_install or pip, with or without administrative privileges:


$ pip install LFPy --user # or
$ sudo pip install LFPy


If LFPy was previously installed, add the --upgrade flag to force reinstall or upgrade.

#### 5.2.2. Installation from source

The LFPy sources can be obtained either by downloading official releases, or by checking out the latest development code from the repository with subversion^21^:


$ svn co \
http://bebiservice.umb.no/svn-public/LFPy-release/
trunk LFPy


LFPy can then be installed by executing the supplied distutils setup.py script with or without administrative rights:


$ cd /path/to/LFPy/
$ python setup.py install --user # or
$ sudo python setup.py install


LFPy can also be used from any folder containing the source code by either issuing or adding the following line to the $HOME/.bashrc or similar file:


$ export PYTHONPATH=PYTHONPATH/:/path/to/LFPy/:


The Cython extensions must then be built in-place by issuing:


$ python setup.py build_ext -i


#### 5.2.3. Testing the installation

If the installation finished without error, and other Python requirements are met (see section 5.1), the importing of LFPy using Python or NEURON as the interpreter, should print out some NEURON credits:


$ python -c “import LFPy” # or
$ nrngui -python -c “import LFPy”
NEURON – VERSION 7.3 (736+:19ad148877ff+)…


#### 5.2.4. Unit tests

If the LFPy source code has been obtained, a test suite built using the unittest module can be run as:


$ cd /path/to/LFPy/unittest
$ python testLFPy.py


The script will execute a series of tests, and summarize the results:


…
Ran 25 tests in 28.735 s

OK


The test suite initially calculates the extracellular potentials from a stick neuron with sinusoidal synaptic input applied to one end, obtained by numerically solving the analytical expression for the extracellular potential (Pettersen and Einevoll, 2008), and subsequently compare with results obtained from equivalent LFPy simulations. If the discrepancy between the extracellular potentials from the analytical expression and LFPy simulations is sufficiently small (typically 3 running digits), tests will pass. Other tests check consistency at different time resolutions, and whether the outputs of the different calculation methods (Eqs. 3-5) converge in the far field, i.e., far away from the neuronal source.

### 5.3. Morphology files

The morphology keyword argument for LFPy.Cell should be a .hoc-file similar to those from the export dialog of the Import3D tool or from the 3D Neuron Viewer application at NeuroMorpho.org^22^ (Ascoli et al., 2007), with the full 3D-specification of the neuron. However, the Cell class will also load SWC, NeuroLucida (v1 and v3), and NeuroML^23^ (Gleeson et al., 2010) files using the Import3D tool internally if such files are detected. The procedure loading the morphology also attempt to load a .rot-file alongside the morphology file itself, with default rotation angles typically applied to orient the apical dendritic tree along the positive *z*-axis pointing upwards (using the right-hand rule, the *xy*-plane is the horizontal plane). The morphology is rotated around the center of the soma. A .rot file is a pure text file containing three lines, each telling the rotation angle in radians around each axis, similar to:


x = 4.729
y = -3.166
z = 0


i.e., as generated by the script /examples/create_rot_file.py.

With the cell keyword argument pt3d=True, rotating or repositioning of cell is applied also to the pt3d information within NEURON.

#### 5.3.1. Visualization

While LFPy does not provide specific plotting functionality, a cell may easily be visualized using, e.g., matplotlib and the following code:


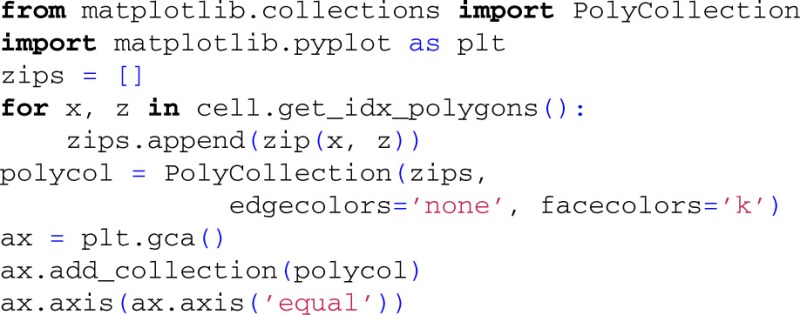


### 5.4. Setting the biophysical properties

The custom_code argument of the Cell-class can be used to pass additional biophysical properties of the model neuron. This argument should be used with HOC-language or Python-statements in .hoc or .py files, respectively. The path to the file should either be provided as strings, or a list pointing a set of files. The files typically contain procedures looping over the sections of the morphology, defining which membrane mechanisms and corresponding densities and properties are present on the section- or compartment level. Another option is to use custom_fun and custom_fun_args arguments of the Cell class to pass regular python functions and optionally arguments for these to set the biophysical properties of the model.

A few example files (e.g., /examples/example5.py and /examples/example6.py) supplied with LFPy, make use of these different methods to specify the biophysical properties of a layer-5 cat pyramidal cell model adapted from Mainen and Sejnowski (1996), by using the relevant parts from the original model code from ModelDB^24^.

Note that the default behavior of the Cell is to insert NEURON's pas and extracellular mechanisms across all sections, and set the number of compartments for each section according to the d_lambda rule, with distance between nodes no longer than a fraction d_lambda=0.1 of length constants λ_*f*_ computed at *f* = 100 Hz (Hines and Carnevale, 2001). This default behavior can be switched off with the Cell keyword arguments:


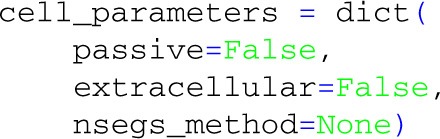


In this case the passive properties, compartmentalization, and optionally the extracellular mechanism to enable calculation of extracellular potentials, *must* be set by the procedures that also set the remaining biophysics of the cell model, otherwise NEURON may fail to assess the equivalent circuit of the model neuron.

If a model loaded in LFPy fails to reproduce the original model behavior, a simple way to verify that properties like temperature, morphology or channel densities are correct is to print and compare the properties of all compartments in both implementations:

In HOC:


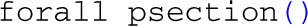


In Python:


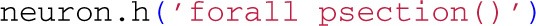


Note that compiled NMODL files present in the working folder will be loaded by default, but such files located elsewhere have to be imported explicitly:


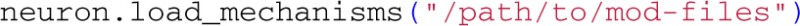


Above, the morphology and specifications of the biophysical properties were given as keyword arguments to LFPy.Cell. Models existing in memory can in principle be executed by supplying the keyword arguments morphology=None, delete_sections=False in addition to the above cell_parameters, e.g., if a model is defined via the NEURON graphical or command line interface. Defining scripts with the full specification of the model loaded with LFPy.Cell, is, however, in most cases more tractable.

### 5.5. Using cell templates

As illustrated in Section 4.2, the TemplateCell class requires a template specification to assign section references. The template specification file should conform to the basic structure of the following example, assigning the somatic, dendritic and axonal sections of a reconstructed morphology to lists:


begintemplate LFPyCellTemplate
public soma, axon, dend, apic
public all, somatic, axonal, basal, apical
objref all, somatic, axonal, basal, apical
proc init() {
    all = new SectionList()
    somatic = new SectionList()
    axonal = new SectionList()
    basal = new SectionList()
    apical = new SectionList()
}
create soma[1], axon[1], dend[1], apic[1]
endtemplate LFPyCellTemplate


### 5.6. The extracellular mechanism and parallel NEURON

LFPy by default inserts the extracellular mechanism provided by NEURON in every compartment (which is useful to simulate the use of e.g., extracellular stimulation electrodes). This mechanism conveniently provides direct access to the total transmembrane current *I_n_*, eliminating the need to individually extract all ionic, resistive, capacitive and synaptic transmembrane currents and sum them. While certain minor limitations for use of the extracellular mechanism together with the parallel capabilities of NEURON provided by ParallelContext exist, i.e., in combination with the multisplit-method and splitcell-method applied to dendritic trees of individual cells (Hines et al., 2008), the present version of LFPy has not been written in a way that exploit NEURONs parallel capabilities. Therefore, LFPy does not support implicit parallel simulations of extracellular potentials with interconnected neurons. Within that scope, different cell-objects in general have to be distributed between different MPI-ranks, and their corresponding connections have to be communicated using the provided ParallelContext interface. Note that in the MPI example in Section 4.3, each cell exists independently without connections on different MPI ranks which does not require the NEURON ParallelContext.

### 5.7. Indexed compartments and section references

LFPy extensively uses numpy array-operations for efficient calculation of extracellular potentials. Most data regarding specific compartments in the model neuron is therefore stored in numpy-arrays with length equal to the total number of compartments in the cell. This includes, for example, the start- and end coordinates of current sources along the *x, y, z*-dimensions and the transmembrane currents in each time step. Each compartment is in this representation assigned a specific (unique) index.

However, the usual way to access compartments in NEURON is through section references and relative coordinates:


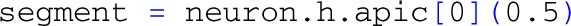


i.e., there are no 'global' indices as in LFPy where instead the compartment indices, idx, correspond to a *counter* in a nested loop over all sections and compartments:


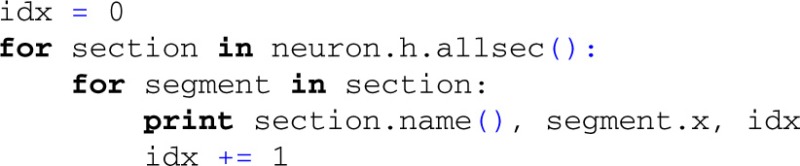


Therefore, to ease this transition for users accustomed to working with specific models in NEURON, LFPy.Cell provides methods for getting indices corresponding to sections in the NEURON namespace


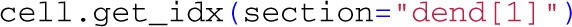


or conversely, getting section names and positions from compartment indices:


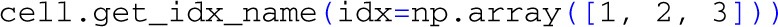


### 5.8. Constructing line sources

Morphologically detailed neuron models resulting from histological reconstruction (De Schutter, 2009), Ch. 8), typically specify sections with an arbitrary number of data points (*x*, *y*, *z*, *d*), i.e., 3D locations and diameters. For a section corresponding to a continuous piece of dendrite split into *N*_seg_ compartments, NEURON creates equivalent cables that correspond to the arc length as specified by the histological 3D-points, where the cables have equal length, but varying diameter, effectively with different electrotonic length constants. The total length of the compartments is equal to the total arc-length of the reconstructed section, but the detailed geometry is not needed for solving the cable equation. Compartments and their respective transmembrane currents must however be assigned a location and orientation in 3D space. In LFPy, the start and end-point coordinates of each straight line source are obtained using linear interpolation along the total arc-length of each section, so that for a section with section.nseg==1 the line source is a straight line between the start and end-points of the section, for section.nseg==2, straight lines between the start, mid- and end-points of each section are used and so forth. As a consequence, the total length of the line sources will typically be less than the total arc length of the section depending on its geometry, but more histological information may be preserved with line sources by increasing the number of compartments per section. This reduction in morphological detail is implied in Figure 2A. The diameter and surface area of each compartment as reported by NEURON is preserved, however, such that the minimum allowable distance between a putative extracellular site and compartment axis will be equal to its radius. If conflicts are detected, the minimum radius employed in extracellular potential calculations is automatically set equal to the respective compartment radius.

### 5.9. Fast and memory-efficient scheme for calculating extracellular potentials

The traditional way of employing the forward formalism for calculation of extracellular potentials as described in section 2.2 is to first extract the membrane currents of a model neuron for the entire duration of the simulation time, and to store them temporarily either in memory or on file (Holt and Koch, 1999; Gold et al., 2006, 2007; Pettersen et al., 2008). The stored transmembrane currents may then be used to calculate the extracellular potentials offline. This is, however, a computationally inefficient and memory-consuming procedure. For example, storing all transmembrane currents of a 1000-compartment neuron model at 64-bit float precision for 1 s of simulation time running at 20 kHz temporal resolution, will require 160 MB of uncompressed binary storage (e.g. with numpy.save() or with HDF5^25^), and as much as ~500 MB in a text file using numpy.savetxt() with %.18e formatting.

As shown in section 4.2, this inefficient and memory-consuming process of intermediate storage of membrane currents can be omitted altogether. Assuming linear superposition of extracellular potentials from different current sources in extracellular media, LFPy can geometrically map the contribution from each compartment-current to any electrode contact point defined with the class LFPy.RecExtElectrode. This is done by temporarily substituting the cell's membrane currents with an (*N, N*) identity-matrix, and running the cell object and corresponding identity matrix through the LFPy.RecExtElectrode class, defining the geometry of an extracellular recording device with *N*_contacts_ contact points. The end result is an (*N*, *N*_contacts_) coefficient matrix **C**, that only needs to be computed once. For simulations of extracellular potentials, the potentials at the different electrode contacts at every time step *t_i_* is then simply given by the dot product

(7) $$\Phi (t_{i})=C\;\cdot \;I(t_{i})\;,$$

where the vector **I**(*t_i_*) of length *N* contains the respective membrane currents, and **Φ**(*t_i_*) a vector with length *N*_contacts_ containing the extracellular potentials at time-step *t_i_*. The memory requirements are potentially reduced by orders of magnitude (≈*N*/*N*_contacts_) vs. in-memory storage of transmembrane currents, at the cost of calculating **C**. Any such array **C**_x_ (or list thereof) can readily be passed with keyword argument dotprodcoeffs to cell.simulate(). LFPy thereby facilitates additional computations relying on compartmental membrane currents, where cell and stimulus can still be used to set up the model.

LFPy will default to storing results in-memory, but simulation of extracellular potentials to HDF5 directly with h5py is supported by setting LFPy.Cell.simulate keyword arguments to_memory=False, to_file=True, and file_name=“some/file/name.h5”.

## 6. Discussion and outlook

We have presented a new Python software package, LFPy, for calculation of extracellular potentials around morphologically reconstructed neurons. Despite its name, the software is not only applicable for calculation of *local field potentials* (LFPs), the low-frequency part of the extracellular potentials (cf. Example 1 in section 3.1). As the biophysical forward-modeling scheme is also applicable for the higher frequencies contained in electrical signals recorded in the brain, LFPy can equally efficiently be used for simulations of high-frequency signals such as extracellular spikes (cf. Example 2 in section 3.2).

While the present version 1.0 of LFPy is focused on the calculation of single-neuron contributions to the extracellular potentials, the computational scheme generalizes directly to the calculation of signals from *populations* of neurons. This was illustrated in Example 3 in section 3.3 where also parallelization of the computational scheme by means of MPI was employed, however without communication between units. At present, LFPy is however less suitable for the investigation of extracellular potentials generated in genuine network models that require parallelization of the network activity. At present, this is a limitation in the current version of the software mainly in that the simulation control is incorporated as an LFPy.Cell class method, and that the class LFPy.TemplateCell (which allows for multiple simultaneous cell representations) is not using the capabilities of NEURON for assigning each cell to different MPI ranks. However, as simulation of extracellular signals from network activity likely will become increasingly important, we aim to implement solutions to these limitations in future versions of LFPy.

The computational scheme presented here, and implemented in version 1.0 of LFPy, is based on the biophysical forward model in Equation (2). This formula inherently assumes an *infinite*, *isotropic*, *homogeneous*, and *ohmic* extracellular medium (Pettersen et al., 2012). However, the scheme can be straightforwardly generalized to account for *anisotropic* conductivities (Nicholson and Freeman, 1975; Logothetis et al., 2007; Goto et al., 2010), or jumps in conductivities at tissue interfaces (Pettersen et al., 2006). Also, even if the conductivity σ is found to be frequency dependent, the forward modeling scheme can still be used for each frequency (Fourier) component separately. For further discussion of the validity and possible generalizations of the present scheme, see Pettersen et al. (2012). Finally, when the extracellular conductivities around the recording electrodes have such a complicated spatial structure that analytical formulas either do not exist or are unpractical [e.g., in cortical slice recordings with multielectrode arrays (MEAs) Bakker et al., 2009], one can always solve the forward problem by means of *finite element modeling* (FEM) (Logg et al., 2012; Ness et al., 2012; Lempka and McIntyre, 2013). A natural avenue of future work is to expand LFPy to account for such new situations as needs arises.

Another natural application of LFPy is the investigation of effects from externally imposed electrical potentials in the extracellular medium, for example due to currents injected during deep-brain stimulation (DBS) (Oluigbo et al., 2012) or induced by transcranial magnetic stimulation (TMS) or transcranial direct current stimulation (tDCS) (McKinley et al., 2012). In fact, externally imposed spatiotemporally distributed extracellular potentials (Anastassiou et al., 2010, 2011; Pashut et al., 2011) may already be included in the present version of LFPy (by use of cell.insert_v_ext()). However, effects from so called *ephaptic* coupling (Anastassiou et al., 2011), where neurons mutually interact via extracellular potentials stemming from their own neuronal activity, is less amenable for investigation by the present version of LFPy.

The development of neuroinformatics tools obviously benefits strongly from an active community of users providing feedback, and possibly even new code reflecting new applications. As we believe that detailed biophysical modeling of extracellular potentials must become an integral ingredient in the future interpretation of such signals, we are hopeful that the present launch of LFPy will signal the beginning of an active community of such modelers, preferably contributing to the joint development of this tool.

### Conflict of interest statement

The authors declare that the research was conducted in the absence of any commercial or financial relationships that could be construed as a potential conflict of interest.

## Footnotes

^1^http://senselab.med.yale.edu/modeldb/.

^2^compneuro.umb.no/LFPy.

^3^numpy.scipy.org.

^4^senselab.med.yale.edu/modeldb/ShowModel.asp?model=2488.

^5^senselab.med.yale.edu/modeldb/ShowModel.asp?model=139653.

^6^www.open-mpi.org/.

^7^mpi4py.scipy.org.

^8^www.python.org.

^9^www.debian.org.

^10^www.cython.org.

^11^docs.python.org/2.7/library/profile.

^12^www.scipy.org.

^13^matplotlib.org.

^14^alfven.org/wp/hdf5-for-python.

^15^mpi4py.scipy.org.

^16^www.ipython.org.

^17^www.neuron.yale.edu.

^18^compneuro.umb.no/LFPy.

^19^www.neuron.yale.edu/neuron/download.

^20^https://pypi.python.org/pypi/LFPy.

^21^subversion.tigris.org/.

^22^www.neuromorpho.org.

^23^www.neuroml.org.

^24^senselab.med.yale.edu/modeldb/ShowModel.asp?model=2488.

^25^www.hdfgroup.org/HDF5/.
